# Polymerase chain reaction-based gene removal from plasmids

**DOI:** 10.1016/j.dib.2015.04.024

**Published:** 2015-05-08

**Authors:** Vishnu Vardhan Krishnamurthy, John S. Khamo, Ellen Cho, Cara Schornak, Kai Zhang

**Affiliations:** Department of Biochemistry, University of Illinois at Urbana—Champaign, Urbana, IL 61801, United States

**Keywords:** Restriction-free cloning, Polymerase chain reaction, Gene removal, Plasmids

## Abstract

This data article contains supplementary figures and methods to the research article entitled, “Multiplex gene removal by two-step polymerase chain reactions” (Krishnamurthy et al., Anal. Biochem., 2015, doi:http://dx.doi.org/10.1016/j.ab.2015.03.033), which presents a restriction-enzyme free method to remove multiple DNA segments from plasmids. Restriction-free cloning methods have dramatically improved the flexibility and speed of genetic manipulation compared to conventional assays based on restriction enzyme digestion (Lale and Valla, 2014. DNA Cloning and Assembly Methods, vol. 1116). Here, we show the basic scheme and characterize the success rate for single and multiplex gene removal from plasmids. In addition, we optimize experimental conditions, including the amount of template, multiple primers mixing, and buffers for DpnI treatment, used in the one-pot reaction for multiplex gene removal.

Specifications table

**Table:** 

Subject area	Biology
More specific subject area	Molecular cloning techniques
Type of data	Table, gel image, text file, figure
How data was acquired	Nanodrop (Thermo Fisher Scientific: Nanodrop 2000C)
	Blue transilluminator (New England Biogroup: NEB-SLB-01W)
Data format	Analyzed
Experimental factors	Plasmids were linearized by inverted PCR with a pair phosphorylated primers and recircularized by blunt-end ligation. A method based on two-step PCR was used to remove multiple gene segments from plasmids.
Experimental features	PCR-based removal of one gene or multiple gene segments from plasmids
Data source location	University of Illinois at Urbana-Champaign, Illinois, USA
Data accessibility	The data are supplied with this article.

Value of the data

• The data provide a method and systematic characterization for removing one gene from plasmids based on inverted PCR and blunt-end ligation.
• The data provide the scheme and characterize the success rate for PCR-based multiplex gene removal.
• The data provide the optimization of methods, including the amount of parent template, multiple primers mixing, and buffer conditions for DpnI digestion, to achieve pure products with multiple genes removed from plasmids based on two rounds of PCR.

## 1 Data, experimental design, materials and methods

### 1.1 Materials

The sequences of oligonucleotides (IDTDNA) were summarized in Table 1. T4 ligase was purchased from NEB (Cat. #: M0202S). Phusion DNA polymerase master mix was purchased from NEB (Cat. #: M0531S). DreamTaq PCR Master Mix (2×) was purchased from Fisher Scientific (Cat. #: K1071). T4 polynucleotide Kinase was purchased from NEB (Cat. #: M0201S). 5-Bromo-4-chloro-3-indolyl β-d-galactopyranoside (X-gal, Cat. #: B4252) and Isopropyl β-d-1-thiogalactopyranoside (IPTG, Cat. # I6758) were purchased from Sigma-Aldrich.

### 1.2 Primer design

**Fig. 1:**
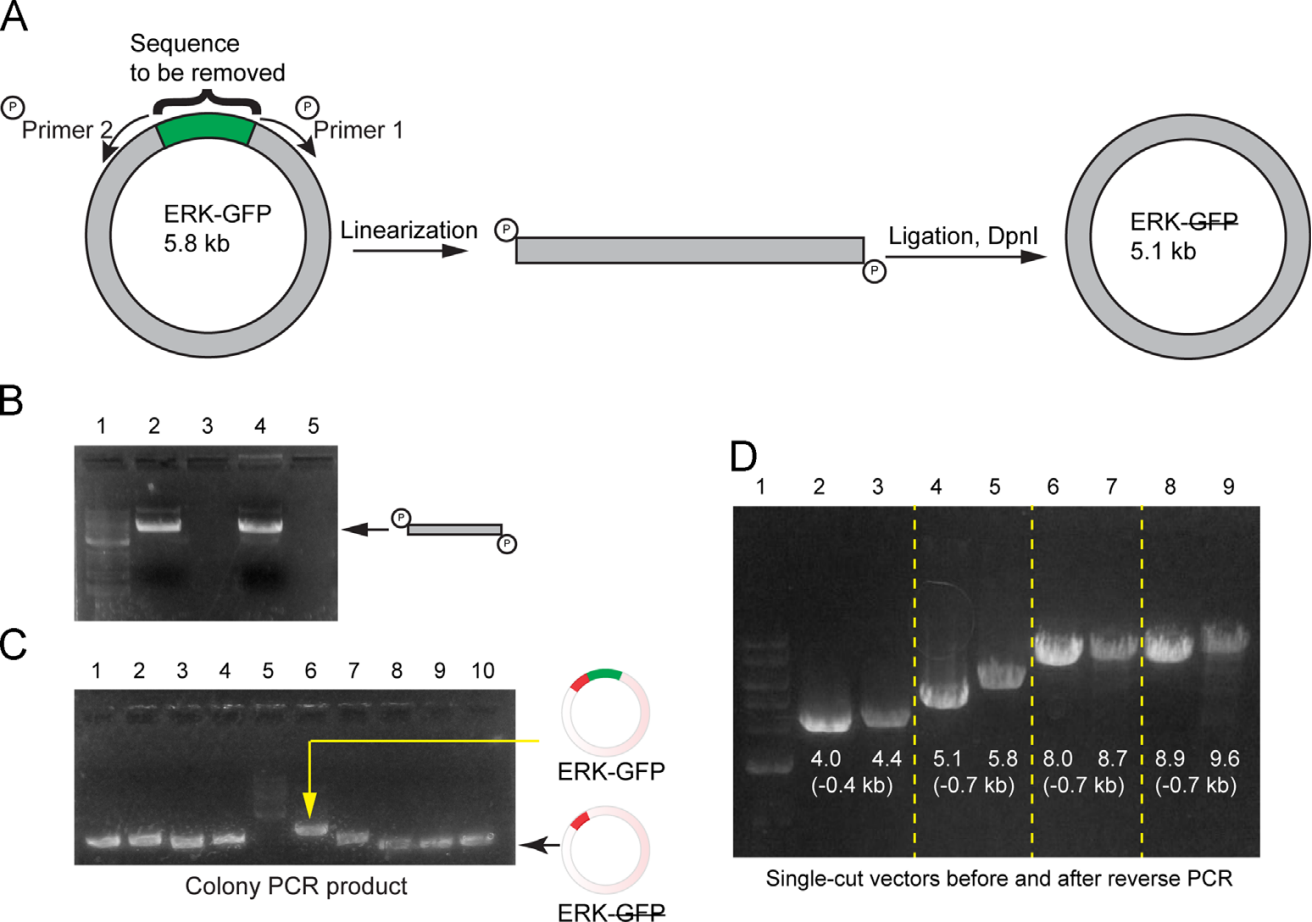
Scheme for one-gene removal by inverted PCR followed by ligation. (A) To remove the *gfp* gene from the ERK-GFP plasmid, a pair of 5׳-phosphorylated primers that excludes *gfp* was used to linearize the plasmid, which was then circularized by blunt-end ligation. (B) Linear ERK-GFP (ERK-GFP plasmid with *gfp* removed). Lane 1: DNA ladder; Lane 2 and 4: linear PCR segments of ERK-GFP from two independent PCR reactions. (C) Colony PCR products of eight colonies (Lane 1–4, 7-10) from ligated ERK-GFP. Products from all colonies migrated faster than that from ERK-GFP (lane 6), indicating successful *gfp* removal in all eight colonies. (D) This assay worked for plasmids with sizes ranging from 4.0 kb to 9.6 kb. After removal of one gene from the vector (the sizes of the genes were indicated in the parentheses), the circular plasmids were amplified. All plasmids (before and after gene removal) were then digested by BamHI, which was a unique cutting site for all plasmids. Successful removal of genes was observed in all four plasmids.

**Fig. 2:**
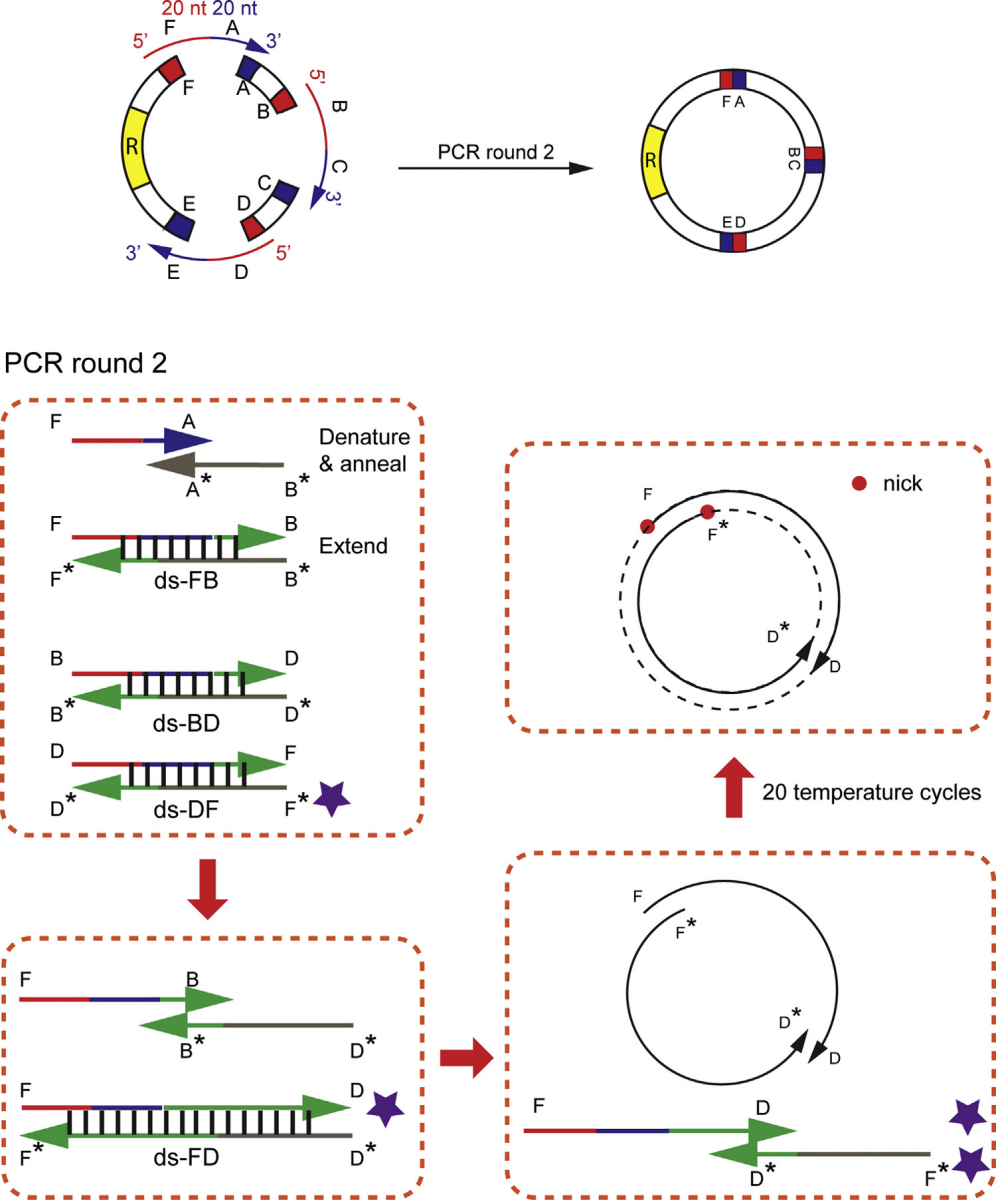
Generation of nicked plasmids in round-2 PCR for multiplex gene removal. One scenario based on a starting single stranded-oligo (ss-oligo), FA, is shown. (Top-left panel) Initial annealing and extension between FA and its template (A^⁎^B^⁎^) generates ds-FB. Similarly, ds-BD and ds-DF can be generated by other ss-oligos. (Bottom two panels) Subsequent annealing and extension between intermediate products generate longer fragments until two annealing fragments have overlapping sequences (20 nt) on both ends (purple stars). (Top-right panel) Nicked circular products are formed after extension. No ligase is needed in this procedure because nicked plasmids can be repaired in cells after transformation.

Single gene removal was achieved by inverted PCR with two 5׳-phosphorylated primers, followed by blunt-end ligation. Multiplex gene removal was achieved by two-step PCR. In such a setting, for each gene segment to be removed, three non-phosphorylated primers were sufficient: two for the first round of PCR and one for the second round of PCR. For the first round of PCR, shared by both single and multiplex gene removal, we designed the primers so that the sense primer overlapped with the downstream sequence of the vector and the antisense primer overlapped with the reverse-complementary upstream sequence of the vector. We varied the length of both primers between 18 and 22 bases in lengths so that their annealing temperatures were within 4 °C of each other. If blunt-end ligation was used, the third primer was not needed. This inverted PCR-ligation method worked well for removing one gene from plasmids with sizes up to 9.6 kb (Fig. 1). For the second round of PCR, each single-stranded oligo was 40 bases in length and had a 20-nt complementarity with the two fragments to be connected [3,4]. Fig. 2 shows one possible scenario for three gene removal based on a specific single-stranded oligo, FA.

### 1.3 Two-step PCR

**Fig. 3:**
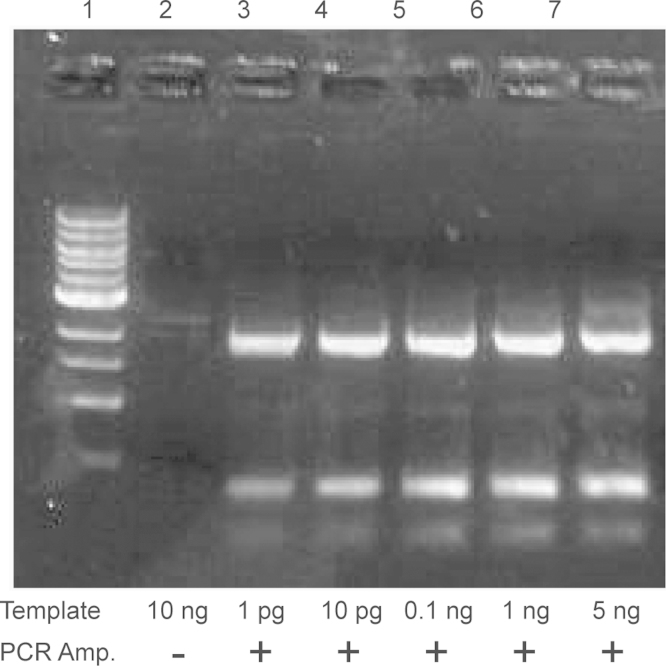
Dose-dependence of the yield of PCR products on the amount of parent template. In a three-gene removal case (*u85*, *f1*, and *kanR*), 1 pg was sufficient to generate enough products. Lane 1: DNA ladder; Lane 2: 10 ng template alone; Lane 3–7: PCR products with 1 pg to 5 ng of template.

**Fig. 4:**
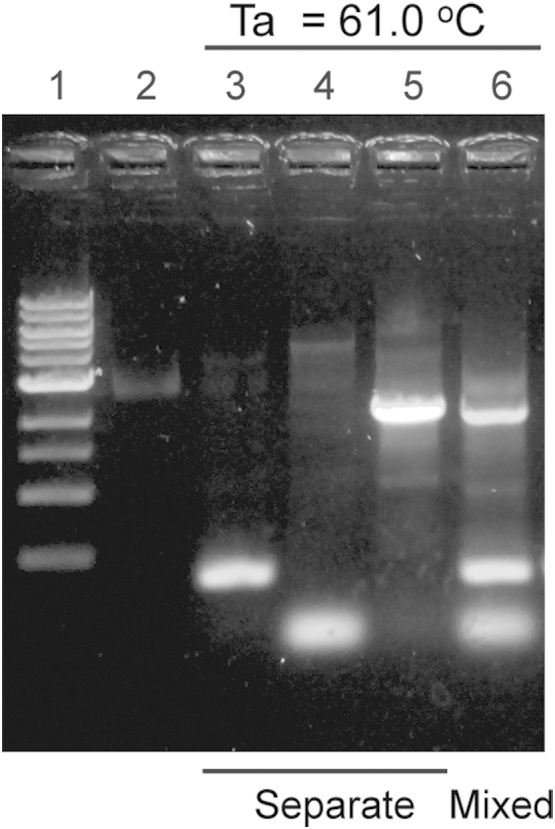
Mixing three pairs of primers did not degrade the quality of linear products compared to separate primer pairs. Ta: annealing temperature. Lane 1: DNA ladder, Lane 2: DNA template alone, Lane 3-5: PCR products with primer pair 1, 2, and 3 in three separate reaction, Lane 6: PCR products with all three pairs of primers mixed in one reaction.

**Fig. 5:**
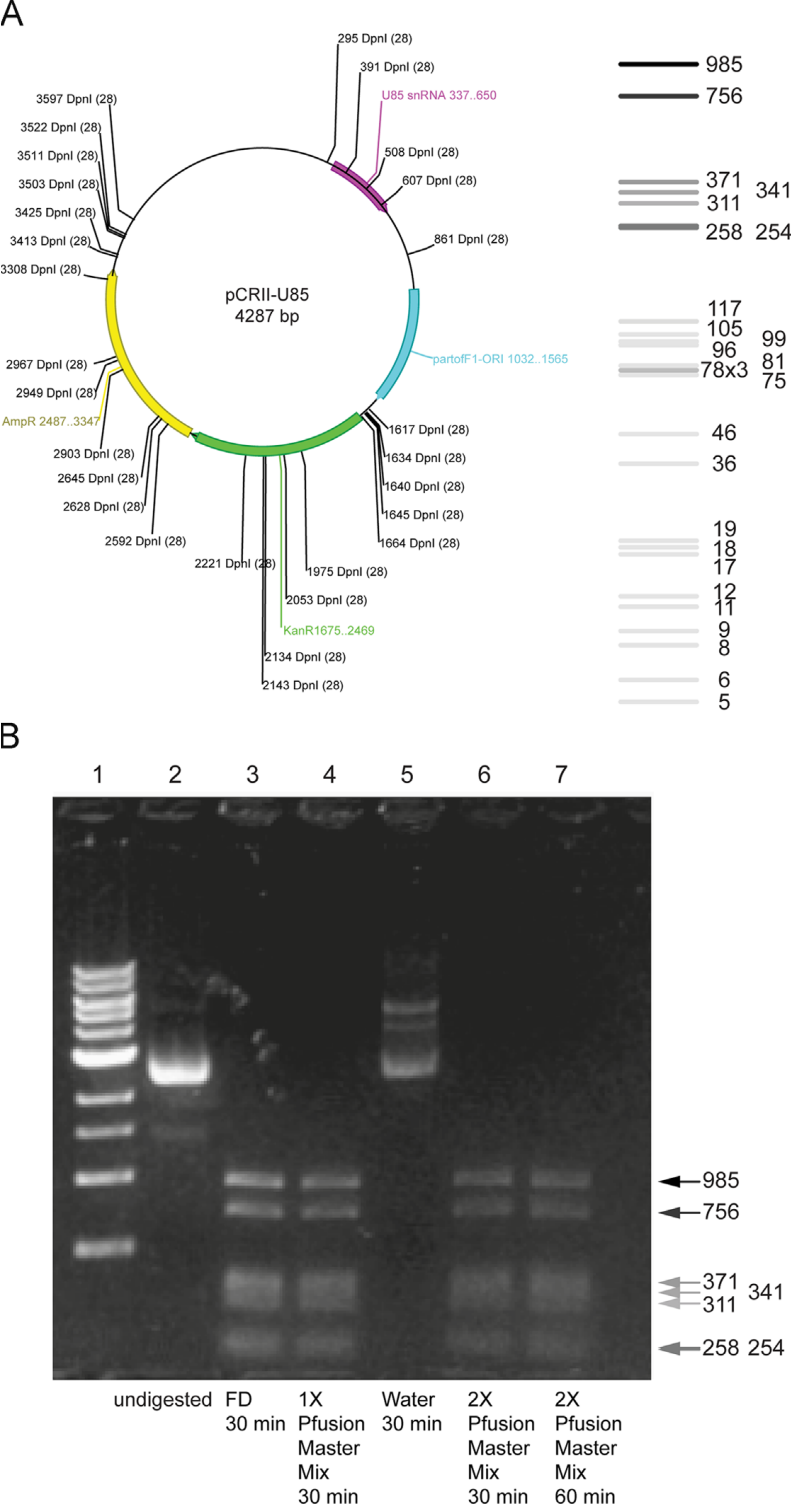
Complete DpnI digestion can be achieved in Phusion master mix. (A)?The graphic representation of pCRII-U85 plasmid marked with four segments and 28 DpnI restriction sites. Purple: *u85*, cyan: *f1*, green: *kanR*, and yellow: *ampR*. The expected DpnI digestion pattern is shown on the right. Both the graphic representation and the digestion pattern were generated by a software named (A Plasmid Editor (APE) developed by M. Wayne Davis), (B)?DpnI digestion of the pCRII-U85 plasmid. A total amount of 400 ng plasmid was used in each reactions. All reactions were performed at 37 °C. Lane 1: DNA ladder, Lane 2: undigested DNA, Lane 3: 30 min digestion in the fast-digestion buffer from the vendor, Lane 4: 30 min digestion in 1× Pfusion Master Mix, Lane 5: 30 min digestion in water, Lane 6: 30 min digestion in 2× Pfusion Master Mix, Lane 6: 60 min digestion in 2× Pfusion Master Mix. (For interpretation of the references to color in this figure legend, the reader is referred to the web version of this article.)

To linearize the vector in round-1 PCR, 10 µl Phusion polymerase 2× master mix (NEB) was mixed with 1 µl of sense and antisense primers (10 µM), 1 µl template (10 pg/µl), and 7 µl of water. In each reaction cycle, the reaction mixture was denatured at 98 °C for 15 s, annealed for 15 s, and extended at 72 °C. The yield of PCR products did not change significantly when the amount of template was adjusted from 1 pg to 5 ng (Fig. 3). When multiple primer pairs were used, the lowest annealing temperature of all primers was used. Mixing three pairs of primers did not degrade the quality of linear products compared to separated primer pairs (Fig. 4). The extension time depended on the longest linear product with 1 kb/min extension rate (e.g. 2 min for a 2 kb linear product). After the reaction, the PCR product was mixed with 2 µl DpnI (10 U/µl) and incubated at 37 °C for 1 h, followed by PCR clean-up. No buffer exchange was required for DpnI treatment, because enzymatic activity of DpnI did not degrade in the PCR master mix of Phusion DNA polymerase (Fig. 5). The final concentration of the fragments was measured by NanoDrop. A typical concentration of the products ranged from 30 to 50 ng/µl. The linear fragments were then circularized by the second round of PCR extension. To set up the round-2 PCR, 250 ng of linear fragments were mixed with 10 µl Phusion polymerase 2× master mix (NEB), 1 µl of ss-oligos (20 µM each), and water to make a 20 µl reaction mix. In each reaction cycle, the reaction mixture was denatured at 98 °C for 15 s, annealed at 55 °C for 15 s, and extended at 72 °C. The reaction was repeated for 20 cycles. The product was then ready for use in transformation.

### 1.4 Transformation

DH5α (provided by Dr. Sandra McMasters in the cell media facility in UIUC) competent cells were used for transformation. Briefly, 30 µl thawed competent cells were mixed with 5 µl products of blunt-end ligation reactions or two-step PCR and incubated on ice for 30 min. Cells were then incubated at 42 °C for 45 s and transferred back to ice and incubated for another 2 min. Cells were then incubated in 1 ml Luria–Bertani (LB) media at 37 °C with vigorous shaking for 1 h. Two hundred and fifty microliters of cell culture were evenly spread onto agar plates and incubated at 37 °C overnight.

### 1.5 Blue/white colony screening assay

To pre-made LB agar plates, one hundred and twenty microliters of X-gal stock solution (20 mg/ml stock in Dimethylformamide) were added and spread evenly using glass spreaders at room temperature. The plates were incubated at 37 °C for at least 30 min to dry. The recovered competent cells were then plated and the plates incubated at 37 °C overnight [5].

### 1.6 Colony PCR reaction

**Fig. 6:**
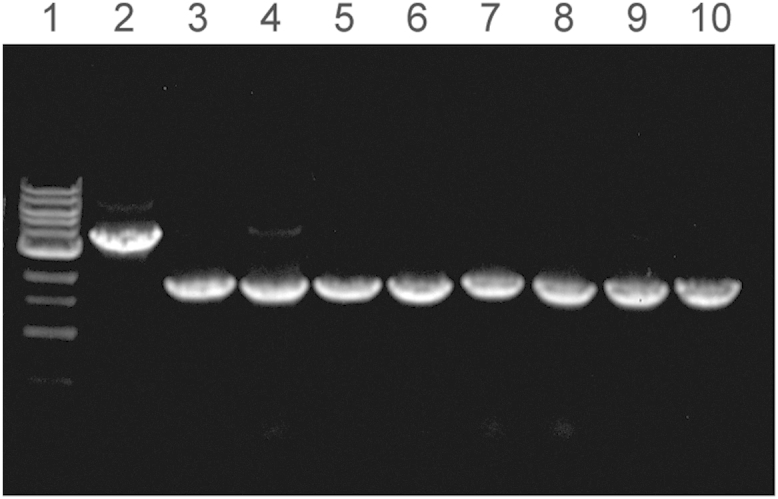
Colony PCR showed that products from 8randomly selected colonies had all three gene segments (*u85*, *f1*, and *kanR*) removed from the plasmid pCRII-U85. Lane 1: DNA ladder, Lane 2: products of full-length pCRII-U85. Lane 3-?10: products of eight randomly selected colonies.

For each colony PCR screening reaction, eight colonies were randomly picked from the agar plate. Each colony was grown in 4 ml LB media in a 14 ml cell culture tube with appropriate antibiotics at 37 °C for 4 h with vigorous shaking, which gave a slightly turbid culture if the colony was successfully transformed. One milliliter of cell culture was then taken from each cell culture tube and transferred to a microcentrifuge tube. The cultures were spun down at 13,000 rpm for 1 min in a mini-centrifuge (Eppendorf). Supernatants were discarded and each cell pellet was resuspended with 50 µl sterile water. Cells were then lysed at 100 °C in a dry heat bath for 5 min and cooled on ice for 2 min. The cell lysates were spun again at 13,000 rpm for 1 min. Two microliters of clear supernatants were used as templates for the following colony PCR (Table 2**)**. After the reaction, products were loaded onto a 1% agarose gel and run at 90 V for 30 min before images were taken on a blue transilluminator. For the experiment of three gene removal, 8 out of 8 randomly selected colonies produced plasmids with the correct size (Fig. 6).

## Figures and Tables

**Table 1:** DNA oligonucleotides used in this study.

**Name**	**Sequence**
phos-ERKrmGFP_for	GGCATGGACGAGCTGTACAAG
phos-ERKrmGFP_rev	CTCGCCCTTGCTCACCAT
PCRIIT_rmU85_for	AAGGGCGAATTCTGCAGAT
PCRIIT_rmU85_rev	AAGGGCGAATTCCAGCA
PCRIIT_rmKan_for	ATTGAAAAAGGAAGAGTATGAGTATTC
PCRIIT_rmKan_rev	GCGAAACGATCCTCATCC
PCRIIT_rmF1_for	TAAGGTTGGGAAGCCCTGCAAA
PCRIIT_rmF1_rev	CGGCGAACGTGGCGAGAAA
ClipU85	GTGTGCTGGAATTCGCCCTTAAGGGCGAATTCTGCAGATA
ClipKan	CAGGATGAGGATCGTTTCGCATTGAAAAAGGAAGAGTATG
ClipF1	CTTTCTCGCCACGTTCGCCGTAAGGTTGGGAAGCCCTGCA

**Table 2:** Colony PCR conditions.

**Material**	**Conc.**	**vol. (µl)**
**DreamTaq Master Mix**	2×	10
**Sense primer (µM)**	25	1
**Antisense primer (µM)**	25	1
**Template (ng/µl)**	10	2
**H_2_O**		6
**Total volume**		20
**PCR program**		

**Step**	**Temperature**	**Time**
**1**	98 °C	3 min
**2**	98 °C	30 s
**3**	58 °C	30 s
**4**	72 °C	*x* min (1.5 min/kb)
**5**	GOTO 2	Rep. 20
**6**	72 °C	10 min
**7**	Hold	4 °C
